# Gene promoter methylation assayed in exhaled breath, with differences in smokers and lung cancer patients

**DOI:** 10.1186/1465-9921-10-86

**Published:** 2009-09-25

**Authors:** Weiguo Han, Tao Wang, Andrew A Reilly, Steven M Keller, Simon D Spivack

**Affiliations:** 1 Wadsworth Center, Human Toxicology & Molecular Epidemiology, Albany, NY, USA; 2 Biostatistics, NYS Dept of Health, Albany, NY, USA; 3 Pulmonary & Critical Care Medicine, Albany Medical College, Bronx, NY, USA; 4 Thoracic Surgery, Albert Einstein College of Medicine, Bronx, NY, USA; 5 Pulmonary Medicine, Albert Einstein College of Medicine, Bronx, NY, USA; 6 Depts. of Epidemiology and Genetics, Albert Einstein College of Medicine, Bronx, NY, USA

## Abstract

**Background:**

There is a need for new, noninvasive risk assessment tools for use in lung cancer population screening and prevention programs.

**Methods:**

To investigate the technical feasibility of determining DNA methylation in exhaled breath condensate, we applied our previously-developed method for tag-adapted bisulfite genomic DNA sequencing (tBGS) for mapping of DNA methylation, and adapted it to exhaled breath condensate (EBC) from lung cancer cases and non-cancer controls. Promoter methylation patterns were analyzed in *DAPK, RASSF1A and PAX5β *promoters in EBC samples from 54 individuals, comprised of 37 controls [current- (n = 19), former- (n = 10), and never-smokers (n = 8)] and 17 lung cancer cases [current- (n = 5), former- (n = 11), and never-smokers (n = 1)].

**Results:**

We found: (1) Wide inter-individual variability in methylation density and spatial distribution for *DAPK, PAX5β *and *RASSF1A*. (2) Methylation patterns from paired exhaled breath condensate and mouth rinse specimens were completely divergent. (3) For smoking status, the methylation density of *RASSF1A *was statistically different (p = 0.0285); pair-wise comparisons showed that the former smokers had higher methylation density *versus *never smokers and current smokers (p = 0.019 and p = 0.031). For *DAPK *and *PAX5β*, there was no such significant smoking-related difference. Underlying lung disease did not impact on methylation density for this geneset. (4) In case-control comparisons, CpG at -63 of *DAPK *promoter and +52 of *PAX5β *promoter were significantly associated with lung cancer status (p = 0.0042 and 0.0093, respectively). After adjusting for multiple testing, both loci were of borderline significance (p_adj _= 0.054 and 0.031). (5) The *DAPK *gene had a regional methylation pattern with two blocks (1)~-215~-113 and (2) -84 ~+26); while similar in block 1, there was a significant case-control difference in methylation density in block 2 (p = 0.045); (6)Tumor stage and histology did not impact on the methylation density among the cases. (7) The results of qMSP applied to EBC correlated with the corresponding tBGS sequencing map loci.

**Conclusion:**

Our results show that DNA methylation in exhaled breath condensate is detectable and is likely of lung origin. Suggestive correlations with smoking and lung cancer case-control status depend on individual gene and CpG site examined.

## Background

Lung cancer is the leading cause of cancer mortality in the U.S. [1]. Most patients will never undergo curative procedures (surgery) because of the wide extent of disease at diagnosis. For earlier diagnosis, screening programs in asymptomatic, high-risk population groups have been studied by several technologies, including cytology of the sputum [2,3], circulating tumor biomarkers [4,5], blood proteomic patterns [6,7], chest tomography [8,9], nuclear magnetic resonance (NMR) [10], and other techniques. Each approach has limited diagnostic specificity as currently applied [11,12], such that identifying particularly high risk individuals for application of these candidate early disease detection strategies may allow leveraging of their performance.

Sampling the target visceral epithelia non-invasively for risk assessment in asymptomatic subjects poses anatomic challenges. Expectorated sputum has been intensively studied for this reason, although up to 30% of current or former smokers do not produce sputum, even after induction with nebulized saline [13-15]. Nonetheless, the successful study of sputum, presumably derived solely from lung epithelia, has been demonstrated in suggestive studies by the New Mexico/Colorado consortium where Belinsky, *et al*. have demonstrated the promise of a multiple gene promoter hypermethylation panel for identifying people at high risk for cancer incidence [14].

Exhaled breath contains aerosols and vapors that can be collected for non-invasive analysis of physiologic and pathologic processes in the lung. To capture the breath for assay, exhaled air is passed through a cooled, condensing apparatus, which is also available as a handheld, disposable device. The result is an accumulation of condensed fluid that is referred to as exhaled breath condensate (EBC). Predominantly derived from water vapor, EBC has dissolved within it aqueous, soluble, nonvolatile compounds. The technique has attracted broad research interest, and there is a significant literature describing its utility in procuring small metabolites for the investigation of inflammatory lung diseases [16,17]. Several investigative groups, including our own, have detected macromolecules in EBC, such as genomic DNA [18-21]. This suggests the possibility of DNA-based analyses of lung processes, including epigenetic alteration.

Promoter hypermethylation is known to cause stable silencing of associated genes and plays an important role in both normal development [22] and disease [23]. Gene promoter hypermethylation is recognized as a crucial component in lung cancer initiation and progression [24]. Most translational studies measuring CpG methylation invoke methylation-specific PCR (MSP) assays that sample 1-4 CpG sites. We recently reported a method for the facile annotation of larger expanses of gene sequence for CpG methylation at single base resolution, using a tag-modification of bisulfite genomic sequencing (tBGS) [21] where all CpG sites could be sampled in a given fragment.

Because of consistent reports as a relevant biomarker class in carcinogenesis, we pursued the appearance of promoter hypermethylation of tumor suppressor genes in a non-invasive exhaled (EBC) matrix putatively representing lung-derived material. In the current study, we analyzed comprehensive DNA methylation maps in EBC from non-cancer control subjects who were never smokers, former smokers, and current smokers, along with a pilot group of incident lung cancer patients, to generate a new non-invasive, epithelial-based method for ascertainment of lung carcinogenesis in humans.

## Methods

### Subjects

A total of 54 subjects (37 non-cancer control subjects and 17 lung cancer case subjects) donated exhaled breath condensate. Thirty six of the first 37 consecutive subjects donated sufficient mouth rinses for anatomic verification for the purposes of this study, in an ongoing lung cancer case-control study. Subjects were of predominantly (>80%) Euro-Caucasian descent, equally women and men, queried on lifetime and proximate smoking habits, as well as medical history and other factors. Questionaire, mouth rinses, and exhaled breath condensate were all sampled prior to any other diagnostic (*e.g*., bronchoscopy) or therapeutic (*e.g*., surgery, chemotherapy) intervention. The procedures followed protocols approved by both the Albany Medical Center, New York State Department of Health Institutional Review Boards, and Albert Einstein College of Medicine Committee on Clinical Investigation (IRB).

Case status was confirmed by conventional positive clinical and histopathologic criteria; for initially negative clinical bronchoscopic biopsies, follow-up biopsy procedures and clinical data were tracked for three months from time of enrollment to affirm the case status. The 17 cases were comprised of six with adenocarcinoma, three with squamous cell carcinoma, five with undifferentiated non-small cell carcinomas, and three subjects with small cell carcinoma. The smoking status of these 17 cancer cases included current smokers (n = 5), former smokers (n = 11), and never smoker (n = 1). The 37 non-cancer controls, with no clinical evidence of cancer at time of enrollment, included current-smokers (n = 19), former-smokers (n = 10), and never-smokers (n = 8). Those control subjects (n = 9) undergoing biopsy of what proved ultimately to be benign nodule were histologically confirmed as controls. The other 28 control subjects were designated as controls by common clinical criteria (no recent suggestive symptoms, or suggestive CXR).

### Exhaled breath condensate (EBC) collection

Exhaled breath condensate (EBC) collection was performed by standard methods. EBC is collected in a handheld, disposable RTube^® ^exhaled breath condenser (Respiratory Research, Charlottesville, VA) which entails a airway valve, inner protective sleeve, outer (cooled to -80°C) aluminum sleeveand insulates, during 10 to 15 minutes of quiet tidal volume breathing, with the exception that subjects were asked to swallow or expectorate all saliva, and to sigh once each minute. Approximately 1.0 ml of EBC was collected from each subject. The collected EBC was stored at -20°C.

### DNA preparation from EBC

From each sample, 0.8 ml of EBC was used for DNA preparation. DNA was prepared with DNA Blood Mini Kit per manufacturer's instructions (Qiagen). We added 5 μg of 60-mer oligo-dT as a DNA carrier to enhance template recovery. DNA was eluted in 55 μl buffer AE (Qiagen). The presence of genomic DNA was confirmed by PCR using 5 μl of sample.

### Bisulfite treatment

Of the EBC DNA extract, 45 μl was used for bisulfite treatment. Bisulfite treatment was performed with DNA methylation kit (Zymo Research), with the reaction condition optimized to 37°C for 3 hours. Finally, DNA was eluted in 10 μl of elution buffer. Non-CpG cytosines were checked for complete conversion to uracils/thymidine in the sequence trace as a positive control, before CpG site data analysis commenced. Samples with any incomplete conversion of non-CpG C's in the sequence trace were to be omitted from further CpG site data analysis; however, there were no cases of incomplete conversion.

### Multiplex PCR

Three sets of gene-specific primers (Table 1) were designed to flank each promoter region of *DAPK, RASSF1A *and *PAX5β*, The multiplex PCR contained 1×buffer (Qiagen, Valencia, CA) with 1.5 mM MgCl_2_, 1 μM of each promoter-specific sense and anti-sense primer, 5 units of HotStar^® ^Taq polymerase (Qiagen) and 5 μl bisulfite-modified EBC DNA. PCR conditions were: 95°C for 15 min, then 5 cycles of 95°C for 10 sec, 52°C for 30 sec, 72°C for 1 min, and 35 cycles of 95°C for 10 sec, 49°C for 30 sec, 72°C for 1 min, and finally 7 min at 72°C. The PCR thermal profiles were programmed into a Perkin-Elmer 9700 thermocycler. The presence of amplicons was confirmed by electrophoresis on a 1.5% agarose gel. In many samples, only one (27.8%) or two (35.2%) of three bisulfite treated amplicons could be detected.

**Table 1:** PCR primers

Multiplex PCR primers	Sequence	Product
RASSF1A-F RASSF1A-R	TTAGTAAAT(C/T)GGATTAGGAGGGTTAG CCACAAAAC(A/G)AACCCC(A/G)ACTTCAAC	325 bp (-254~+70)

DAPK-F DAPK-R	AGGGTAGTTTAGTAATGTGTTATAG ACCCTACC(A/G)CTAC(A/G)AATTACC(A/G)AATC	391 bp (-312~+78)

PAX5β-F PAX5β-R	GAGTTTGTGGGTTGTTTAGTTAATGG-3' AACAAAAAATCCCAACCACCAAAACC-3'	322 bp (-147~+174)

tBGS Primer		

RASSF1A-TF RASSF1A-TR	CGACTCCTGCACTCATTAACCCTCACTAAAGAGGGT(T/C)GGATGTGGGGATTT GGCCAGTGAATTGTAATACGACTCACTATAGGGAGGCGGCCCAAAATCCAAACTAAAC	337 bp (-254~+39)

DAPK-TF DAPK-TR	CGACTCCTGCACTCATTAACCCTCACTAAAGTGGGTGTGGGG(T/C)GAGTGGGTG GGCCAGTGAATTGTAATACGACTCACTATAGGGAGGCGGCTCC(A/G)C(A/G)AAAAAAACAAAATC	358 bp (-240~+50)

PAX5β-TF PAX5β-TR	CGACTCCTGCACTCATTAACCCTCACTAAAGGTTATTTTGATTGGTTTGGTG *GGCCAGTGAATTGTAATACGACTCACTATAG*GGAGGCGGCTACC(A/G)AAACTAAAATAAAAC	301 bp (-92~+141)

	Quantitative MSP primers	

DAPK-qF DAPK-qR	AG(C/T)G(C/T)GGAGTTGGGAGGAGTA CAAAC(A/G)ACCAATAAAAACCCTACAAAC	121 bp (-179~-58)

Probe		

DAPK-P1m	VIC-AACGAACTAACGACGCGA-MGB	-99 - -82

DAPK-P1u	6FAM-TACAAACAAACTAACAACACAA-MGB	-99 - -78

DAPK-P2m	VIC-CTACGCGACGCTCGC-MGB	-158 - -144

DAPK-P2u	6FAM-AATTCTACACAACACTCACT-MGB	-159~-140

All gene sequences are from Human Genome sequence using NCBI sequence viewer v2.0. Primer sequences displayed in 5' to 3'end. Italic letters are tag sequence and the underlined is sequencing primer.

### GC tag-modified bisulfite genomic DNA sequencing (tBGS)[21]

The multiplex PCR products were used as template (1 μl) and re-amplified by GC-tagged primers separately (Table 1). The PCR conditions were: 95°C for 15 min, and 5 cycles of 95°C for 10 sec, 50°C for 30 sec, 72°C for 1 min, 30 cycles of 95°C for 10 sec, 65°C for 30 sec, 72°C for 1 min, and finally 7 min at 72°C. PCR products were then purified with a Gel Extraction Kit (Qiagen) and subjected to direct-cycle sequencing on a Perkin-Elmer Biosystems ABI model 3700 automated DNA sequencer, using tag-targeted sequencing primers: 5'-ATTAACCCTCACTAAAG-3' (Forward); 5'-AATACGACTCACTATAG-3' (reverse). Manual review of sequence chromatograms containing two peaks at any one CpG locus was performed by measuring the peak height of the C (or anti-sense G) versus the combined height of the C+T peaks, and generating a C/C+T (or anti-sense A/A+G) peak height representing the methylated fraction of DNA molecules at that CpG site, as a percentage [25,26].

### Quantitative methylation-specific PCR (MSP)

In order to (a) complement the sensitivity limits inherent to sequencing-based technologies such as tBGS, (b) to replicate CpG site sampling approaches used in the literature, and (c) to provide independent corroboration of technical feasibility of exhaled DNA methylation analyses, we analyzed a consecutive subset of 36 available EBC specimens (16 current smokers, 9 former smokers, 7 never-smokers, and 4 lung cancer patients) from the initial 37 EBC samples, using quantitative MSP. Two sets of MSP probes were used. Probe 1 (Table 1) was specific for -82 to -99 (a low methylation region by tBGS), and probe-2 specific for -144 to -158 (a high methylation region by tBGS).

Quantitative MSP for *DAPK *promoter was performed on an ABI Prism-7500 realtime thermocycler, using a 96-well optical tray with caps at a final reaction volume of 20 μl. Samples contained 10 μl of TaqMan^® ^Universal PCR Master Mix, No AmpErase^® ^UNG (uracil-N-glycosylase), 1 μl of 1:1000 diluted multiplex PCR product, an additional 2.5 U of AmpliTaq Gold (Perkin Elmer), 2.5 μM each of the primers and 150 nM each of the fluorescently labeled probes for methylated and unmethylated templates. The specificity of each probe was confirmed by positive and negative control templates, and water blanks. The cloned *DAPK *promoter methylated with CpG methyltransferase was used as positive control included in all experiments. To generate a standard curve, we prepared different ratios of methylated *versus *unmethylated target sequences by mixing methylated and unmethylated DNA. The following ratios were prepared (methylated/unmethylated): 0/100, 10/90, 20/80, 30/70, 40/60, 50/50, 60/40, 70/30, 80/20, 90/10, 100/0. To verify whether MSP sampling probes, targetting variable regions of methylation, would indicate discordant patterns of MSP-designated methylation, we designed two spatially separated sets of probes for the *DAPK *promoter, one in a 5' upstream, tBGS-defined high methylation region (adjacent to CpG residue -158), and one in a 3' downstream low methylation region (adjacent to CpG residue -99) (Table 1). Results were verified by gel electrophoresis of the PCR product. Correlations were made between qMSP and tBGS results at the relevant two target loci, by correlating the percent methylation determined by the respective MSP probe, with the fraction of sites found methylated by tBGS at that same four-CpG MSP site locus (where individual CpG sites were generally dichotomous as methylated or not).

### Data analysis

The tBGS-generated CpG methylation sequence chromatogram tracings data were converted to dichotomous data at each CpG site, where >20% C/C+T peak height ratio by sequence trace was considered methylated, and <20% ratio was considered unmethylated, as the limits of detection for the technology are 5-10% methylated/total DNA molecules, at any given CpG site. Methylation density was defined as the methylated CpGs divided by total CpGs examined in a gene promoter in a given sample. The methylation densities among smoking groups and case group were evaluated by ANOVA and the position specific CpG methylation state was tested for correlation substructure, and then tested by Fisher's exact test. Further tests on each CpG locus within each promoter region were performed by logistic regression [27,28]. Correlations between the qMSP data and tBGS data at the two respective probe loci were tested by Pearson product moment analysis.

## Results

### Reproducibility of DNA methylation mapping in EBC

**Figure 1:**
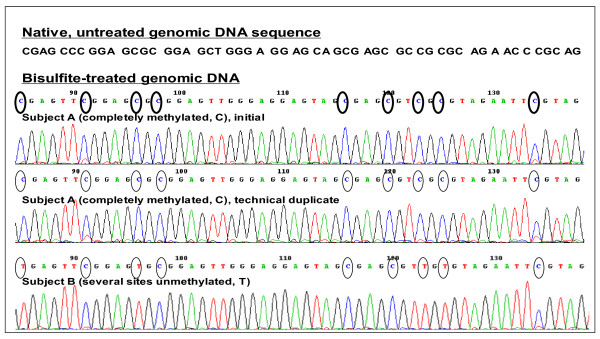
**Tag-adapted sequencing chromatograms from exhaled breath condensate**. For a portion (~250 bp) of the *DAPK *promoter region just 5' to the transcription initiation site (TIS), displayed for two representative subjects A and B. *Top two tracings*: Subject A (all CpG sites methylated, circled C's). The two top tracings are technical replicates from PCR to sequencing for this subject. *Bottom tracing*: Subject B (several CpG sites unmethylated, circled Ts). Detection of partial methylation at a given site is also feasible.

**Figure 2:**
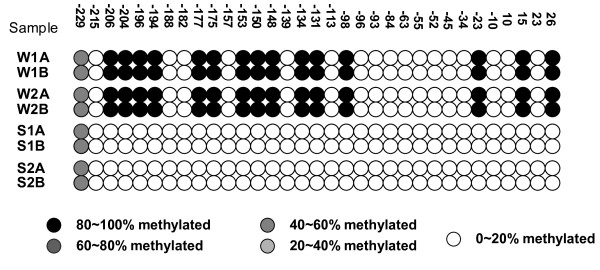
**Reproducibility of DAPK promoter methylation mapping in EBC**. Each of two subjects (W and S) had two consecutive 10-minute EBC collections (1 and 2) separated in time by one hour. Displayed is the tBGS map readout from each of these separate samples, additionally performed as technical replicates (A and B). Both temporal and technical replicates are identical, for a given individual. Methylation density is the simple count of methylated CpG sites (W1A and W1B = 16) over total CpG sites (=33), here yielding 48.5%.

To initially test the reproducibility of DNA methylation mapping in EBC, we collected two consecutive EBC samples, separated in collection time by two hours, from each of two individuals. Each EBC sample was split into two technical replicates for *DAPK *promoter methylation mapping, and these technical and temporal replicates were assayed. The results show that the methylation pattern is completely consistent within samples as technical replicates, and across this brief two hour time period as temporal/biological replicates, for each individual (Figures 1 and 2). There were no episodes of incomplete cytosine conversion, using our protocol, within the 95% sensitivity/resolution limits inherent to sequencing-based chromatographic technologies.

### Origin of exhaled DNA

**Figure 3:**
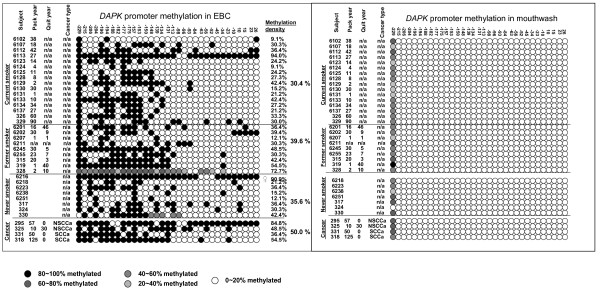
**Comparison of Methylation mapping of *DAPK *promoter in exhaled breath and mouthwash-exfoliated DNA**. (a): Methylation mapping of exhaled breath DNA. (b) Methylation mapping of mouthwash-exfoliated DNA. Exhaled breath condensate (EBC) from 37 of 38 initially recruited consecutive donors and available mouthwash from 36 of the 37 EBC donors, was screened using the tBGS multiplex technique for simultaneous assay of three gene promoters' CpG islands within ~200-300 bp surrounding the TIS. Only mapping results for the *DAPK *promoter are shown. Subject historical smoking features are listed on the left. Mean percent of sites methylated is listed by smoking and case strata, in larger font, on the right. Wide inter-individual methylation variability within any given smoking stratum is apparent. All samples are collected prior to any diagnostic or therapeutic procedure.

To help verify that EBC-DNA is predominantly derived from the lower airway, we reasoned that methylation patterns themselves might differ between epithelia, conferring the expression features unique to those epithelia. We therefore compared the methylation pattern of *DAPK *in paired EBC and mouthwash samples from the initial recruitment set of 37 consecutive subjects with adequate amounts still available from both specimens in 36 of the 37 donors. Results showed that *DAPK *methylation pattern in mouthwash is largely unmethylated, except for the first position CpG site, and therefore completely divergent from that in exhaled breath (Figure 3).

### Promoter methylation mapping across genes and subjects

Of the five initial genes selected for evaluation (*DAPK, RASSF1A, PAX5β, CDH1, p16*) based on their literature reported, methylation-specific PCR (MSP)-based prevalence in lung tumors (>25%), diversity of function, and timing for inactivation during lung cancer development, where known, we chose to pursue the three that showed any promoter methylation at all. We mapped the promoter methylation status of each gene by tBGS.

**Figure 4:**
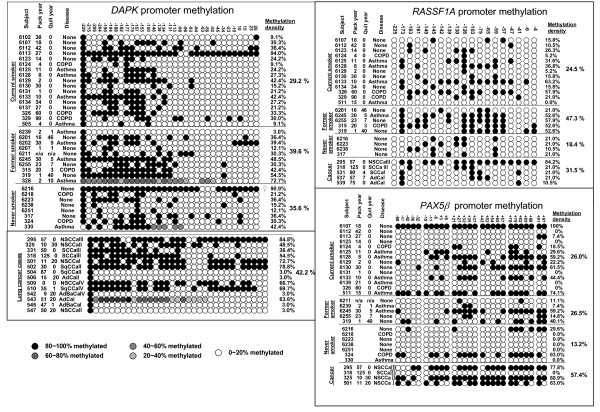
**Methylation maps of *DAPK, RASSF1A, PAX5β *promoter from Exhaled Breath Condensate**. The promoter methylation status of DAPK, RASSF1A, PAX5β, was mapped using tBGS. Overall, both the methylation density and patterns of *DAPK, RASSF1A or PAX5β *promoters differed quite dramatically between individuals within any given smoking or clinical stratum. Methylation density is given at right, for individuals and group means. [NSCCa: non-small cell lung cancer: SCCa: Small cell lung cancer; SqCCa: squamous cell lung cancer; AdCa: Adenocarcinoma; AdBaCa: Adenocarcinoma with bronchalveolar features]. Data on smoking status (never, former and current), pack year, quit years and for tumors, histology and stages I, II, III, IV are given at left.

Overall, the methylation density and patterns for the three promoters (*DAPK, RASSF1A *and *PAX5β*) differed quite dramatically between individuals (Figure 4), otherwise not readily explained by differences in pack-years, quit years, and other factors (below). There were, for example, high methylation outlier individuals apparent (*e.g*., the methylation density of *DAPK *in subject 6113, male current smoker, 27 pack-years, is 96%; Subject 6216, female never smoker, is 91%).

### Promoter methylation density in non-cancer controls

**Figure 5:**
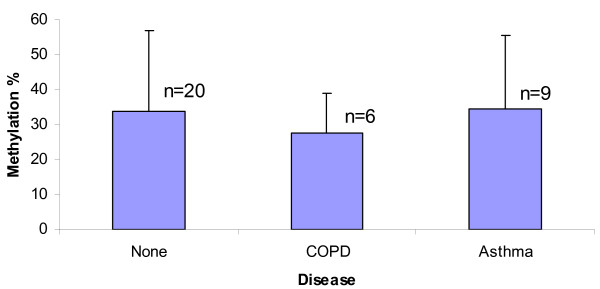
**Methylation density of *DAPK *promoter in non-cancer controls by underlying lung disease**. The methylation density of *DAPK *promoter in EBC samples from COPD, asthma and non-lung disease donors was compared by ANOVA multiple group comparison. There was no significant difference in methylation density between asthma, COPD and the non-diseased group (n = number of subjects). (p = 0.806)

EBC samples from 37 non-cancer controls were analysed by tBGS, and included samples from 11 subjects with asthma, 6 with COPD and 20 non-diseased subjects. In initial univariate analyses of EBC methylation, inclusive of all three methylated promoters, there was no significant difference in the overall methylation densities. However, the methylation density of *RASSF1A *was statistically different between smoker and nonsmoker group (p = 0.0285) and the differences between former versus never smokers and former versus current smokers were also significant (p = 0.019 and p = 0.031, resp.)(Table 2). We also analyzed DAPK promoter methylation *versus *underlying lung disease type in controls. There was no significant difference in methylation density between asthma, COPD and the non-diseased group. (p = 0.806, Figure 5).

**Table 2:** Methylation densities among smoking groups

	Methylation density (SD)			
		
Subjects (n)	*DAPK*	RASSF1A*	*PAX5β*	Pooled
Never smoker (8)	0.365(0.248)	0.184(0.0526)	0.132 (0.246)	0.240(0.168)
Current smoker (19)	0.294(0.198)	0.232(0.19675)	0.296 (0.328)	0.251 (0.153)
Former smoker (9)	0.377(0.211)	0.474(0.149)	0.244 (0.248)	0.374 (0.220)

*p = 0.02854 (Former vs Never: p = 0.019; Former vs Current: p = 0.031)

We further examined each CpG of the *RASSF1A *promoter region using Fisher's exact test. There were five positions with significant differences between former and never smokers (-173, -103, -79, -65 and -57) and three positions between former and current smokers (-173, -79 and -65). After adjusting for multiple testing using a permutation procedure, only two positions (-173 and -65) were significantly different between former smoker and never smokers (p = 0.0079, p_adj _= 0.031)

Methylation density of *DAPK, RASSF1A *and *PAX5β *in controls appeared to be increased with age, but this was not statistically significant. Pack-years, diet, and occupational risk in controls also did not show association with methylation densities in this small pilot analysis.

### Promoter methylation density in lung cancer cases

**Figure 6:**
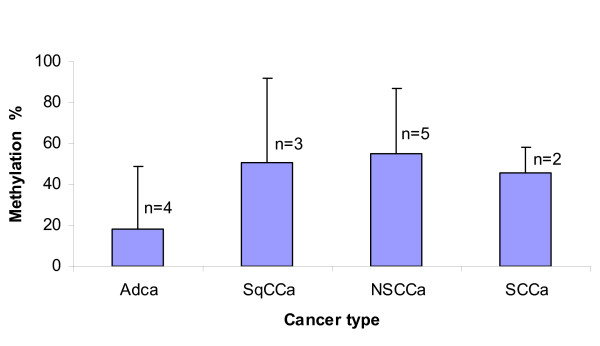
**Methylation density of *DAPK *promoter by tumor histology in lung cancer cases**. The methylation density of *DAPK *promoter in EBC samples from adenocarcinoma, squamous cell carcinoma, non-small cell carcinoma and small cell carcinoma (n = number of subjects) was compared by ANOVA multiple group comparison. There was no significant difference in methylation density between adenocarcinoma, squamous cell carcinoma, non-small cell carcinoma and small cell carcinoma (p = 0.401)

**Figure 7:**
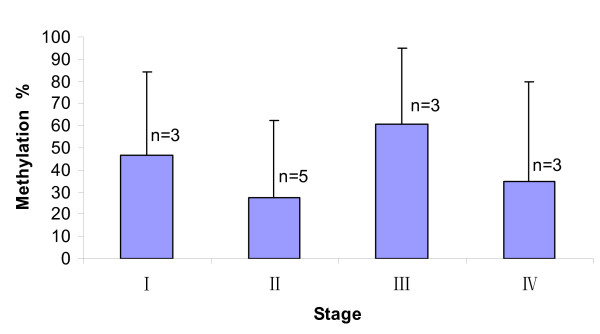
**Methylation density of *DAPK *promoter by stage in cancer cases**. The methylation density of *DAPK *promoter in EBC samples from different stages of lung cancer was compared by ANOVA multiple group comparison. There was no significant difference in methylation density between lung cancer stages(n = number of subjects) (p = 0.728).

While it appeared that methylation densities in cases appeared higher than those in controls in promoters of three candidate gene, global patterns were not statistically significant (Table 3). In more localized tests on each CpG locus within each promoter region, CpG at -63 of *DAPK *promoter and CpG at +52 of PAX5β promoter were significantly associated with lung cancer versus non-cancer controls (p = 0.0042 and 0.0093, respectively). After adjusting for multiple testing, both loci were at the borderline of significance (p_adj _= 0.054 and 0.031). We also analyzed the *DAPK *promoter methylation for tumor histology and clinical stage effects in cases (Figure 6, 7). There was no significant difference in methylation density among tumor histologies (p = 0.401, Figure 6) nor among stages of non-small cell cancer (p = 0.728, Figure 7).

**Table 3:** Methylation density in lung cancer cases *versus *controls.

	Methylation density (SD)			
		
Subjects (n)	*DAPK*	*RASSF1A*	*PAX5β*	Pooled*
Lung cancer (17)	0.422 (0.326)	0.316(0.298)	0.574 (0.397)	0.369(0.312)
Non-Cancer (37)	0.332(0.208)	0.285(0.196)	0.236 (0.288)	0.277 (0.176)
Total (54)	0.358(0.247)	0.292(0.213)	0.280 (0.318)	0.306 (0.229)

*p > 0.05.

### Regional methylation pattern analyses

**Figure 8:**
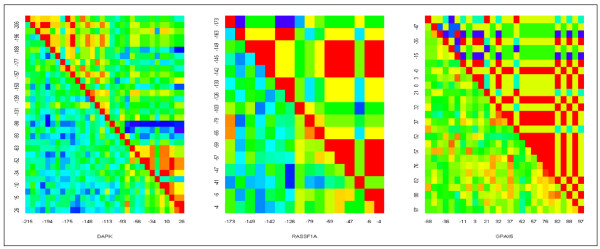
**Positional correlation substructure of EBC methylation in the three promoters**. The non-independence of the positions (clustering of CpG sites that are methylated appears to be non-random, for both cases and controls) suggested a different statistical analytic technique. The *DAPK *controls lower left, leftmost panel) shows mild grouping sufficient to define two regions (about -215 --- -113 and -84 --- +26 near the transcription initiation site). For all cases (upper right of diagonal) and *RASSF1A *and *PAX5β *controls (lower left) there is no apparent no clear grouping by region. The gradient goes from blue (no correlation, r = 0) to green to yellow to red (complete correlation, r = 1.0)

We examined correlation substructure by position, to reveal any clustering or spatial patterns using logistic regression (Figure 8). The *DAPK *promoter uniquely appeared to have a regional methylation pattern with two blocks (block 1: -215~-113 and block 2: -84~+26), in which different CpG positions tend to have similar methylation status. Applying logistic regression on methylation density for each block, we found cases and controls had similar methylation density in block 1, but were significantly different in methylation density in block 2 which lies near the transcription initiation site (p = 0.045) (Table 4).

**Table 4:** Regional methylation pattern of *DAPK *promoter

	Regional methylation of *DAPK *promoter (SD)	
	
Subjects (n)	Block 1 (-215~-113)	Block 2 (-84~+26)
Lung cancer (17)	0.546 (0.387)	*0.304(0.389)
Non-cancer (37)	0.521(0.272)	0.110(0.220)

*:p < 0.05

### Quantitative MSP analysis of DAPK promoter

To analyze the EBC specimens with a second method, for corroboration, quantitative MSP was performed, for the 33 EBC samples available after the primary tBGS mapping assay was complete. We employed two sets of probes for two different locations in the *DAPK *gene: Probe 1 was specific for downstream positions -82 to -99 (a low methylation region as previously assayed by the tBGS assay); and Probe 2 was specific for -144 to -158 (a high methylation region as previously assayed by the tBGS assay). First, the results again indicated DNA methylation analyses are feasible in exhaled breath, by this second assay technique. Second, the qMSP results correlated with those of tBGS at the same loci (Probe 1, r = 0.523, p = 0.00427; Probe 2, r = 0.538, p = 0.00313). Third, the MSP results from Probe 1 were divergent with those from Probe 2 (r = 0.329, p > 0.05), indicating that methylation status in any one annealing site location, could not readily be inferred from that of another site, even when closely spaced or adjacent.

## Discussion

The results of this study show that: (a) measurement of DNA methylation in exhaled breath condensate is feasible; (b) the DNA appears to be of lower airway or lung origin; and (c) has some association with lung cancer and smoker status, depending on gene and individual CpG site examined.

It has long been clear that the gas phase of exhaled breath, and the aqueous condensate phase, contains small molecules that can be analyzed for pathologic processes in the lung, such as for asthma. For larger molecules, such as DNA-based studies, both Gessner et al. [18] and Carpagnano et al [19,20] have demonstrated the possibility of detecting DNA-based sequence alterations in EBC from patients with non-small cell lung cancer. We confirmed that ability, and further optimized the collection and DNA extraction procedures. We then adapted a bisulfite conversion approach and developed two-step nested PCR amplification, while limiting multiplexing, to allow for consistent analyses of these trace specimens, in a recently-devised and comprehensive methylation mapping assay [21].

Our results showing the complete discordance between the respective exhaled and mouthwash DNA methylation map "fingerprints" implies that the predominant origin of exhaled DNA was not contamination from the mouth. Indeed, if mouth-derived DNA is present in EBC, it should be less than 10% of total DNA in EBC. This conclusion is based on the: (1) sensitivity limits of tBGS (>10%) that preclude complete exclusion of mouth derived (unmethylated) DNA in EBC at CpG sites that show methylation; and (2) the detection of a negative (unmethylated) signal could potentially be subsumed in the positive signal at methylated sites, although a review of the sequence tracings did not bear this out. The precision limits of the semi-quantitation afforded by sequence chromatograms for partial methylation (intervals of ~20% intervals), were previously published [21] and appear as shades of gray, in the maps. This initial study therefore suggests that the largest proportion of EBC derives from the lower airway, as judged by the fact that exhaled specimens are discordant from the mouthrinse specimens in methylation pattern, when collected from the same individuals, for the one gene promoter (*DAPK*) so tested. We have ongoing studies more directly addressing the anatomic origin of exhaled DNA, by direct bronchial brush and bronchoalveolar lavage methylation comparison to EBC methylation from the same donors.

Critical to the development of a marker panel for early detection of lung cancer is the selection of genes whose methylation is common but occurs during different stages of lung cancer development. In this study, three genes (*DAPK, RASSF1A *and *PAX5β*) showed methylation among the five candidate genes originally selected. While the *p16 *gene methylation has been reported as one of the earliest methylation events in lung cancer development, occurring in the bronchial epithelium of some current and former smokers [29], we did not find methylation in pretested exhaled samples, nor in the lung cancer cell line A549 cells (not shown). This may be because of the 5-10% sensitivity limitations of tBGS and/or for A549 cells, cell line differences that may not reflect tumor markers. The vast majority of published data has employed some form of methylation specific PCR, which is much more sensitive than sequencing based tBGS for methylation at a given CpG site, by perhaps 10-100-fold. It should be noted that this relative insensitivity of tBGS for methylation at any given site, but broad coverage of multiple CpG sites that may bear on expression, is suitable for many situations where minor degrees of methylation at isolated sites may not be biologically relevant, as the ultimate promoter readout is functional gene expression.

We chose commonly studied tumor suppressor genes such as *DAPK*, and *RASSF1A *precisely because they had been reported to be later events in lung cancer. Indeed, methylation of the *DAPK *and *RASSF1A *genes is uncommon (3% and 0%, respectively) in bronchial epithelium from smokers without cancer, using MSP-based methods [29]. Nonetheless, our bisulfite sequencing results showed the methylation density of *RASSF1A *was statistically different between smoker and nonsmoker group (p = 0.0285). Methylation of *DAPK *has been detected in alveolar hyperplasias in a murine model of lung adenocarcinoma, supporting a role for this gene in the progression of carcinogenesis [30]. The *PAX5β *gene function appears to entail nuclear transcription factors important for cellular differentiation, migration, and proliferation [31], and methylation is reportedly altered in lung tumors. With work on technical limitations to multiplexing underway in this laboratory, we envision an expanded geneset for more comprehensive assessment of the utility of exhaled DNA methylation biomarkers in classifying phenotypes, and ultimately, assigning the risk status of the epithelium.

Initial DNA methylation mapping projects illuminate both the complex distribution of DNA methylation in the human genome, and the importance of inter-individual variation among DNA methylation profiles from different individuals [32-34]. The complexity of methylation map patterns in EBC suggests that comprehensive promoter methylation mapping may be more reflective of the methylation state of a promoter than probe-based methods that sample only 1-4 sites in aggregate, such as MSP. And while chance is possible, the site-specific detail or clustering patterns of more comprehensive methylation map patterns (*e.g., DAPK*) may have specific regulatory consequences, particularly when considering broader regions of a gene promoter. Functional studies approaching this hypothesis are ongoing in the laboratory. Such functional studies would be important for optimizing cancer biomarker identification for robustness and precision; and for targeting by genetic or small molecule interventions.

The quantitive MSP analyses of *DAPK *using two spatially separated probes did show the discordance between methylation at the two designated sites that had originally been mapped as discordant by tBGS. This reinforces the idea that (a) tBGS data is generally concordant with MSP data, based on CpG sites where both assays have been applied; and (b) inference of methylation from one CpG site or region to another is fraught with uncertainty. Additionally, the reasonable correlation between the quantitive MSP and tBGS findings, at each of the two probe sites, was reassuring to the validity of tBGS mapping in these trace exhaled specimens.

For initial confirmation of control status, each control subject who underwent biopsy for clinical indications did also undergo imaging routinely, prior to consideration of dominant lesion biopsy, per clinical routine. This would exclude a significant "missed cancer", other than the one biopsied. Additionally, any subject undergoing a biopsy procedure that had initially negative clinical bronchoscopic biopsies, follow-up surgical or other biopsy procedures and clinical data were tracked for three months from time of enrollment, to reconfirm control status. For those controls not imaged/biopsied by clinical routine, while control misclassification is always a potential problem in case-control studies where some controls are drawn from an at-risk population, with little prospective follow-up, we feel that the thorough vetting of all available clinical and pathologic data in a three month timeframe after enrollment minimized this potential problem. Clearly, prospective follow-up is needed to definitively ascertain outcome, a good design for future more ambitious biomarker studies.

We do not envision exhaled DNA as a method for detection of a small, peripheral tumor. Rather, as field carcinogenesis progresses over the lung epithelia, transforming cells and their debris containing methylated tumor suppressor genes will be shed, marking an increased probability for a lung tumor to arise somewhere, but likely not directly exfoliating from an existing lung tumor in a given deep anatomic location. The exhaled DNA might better be viewed as a whole lung epithelium sampling tool. Therefore, the performance of this biomarker class in predicting lung cancer (*i.e*. in risk assessment) could be viewed as akin to other "risk factors" for any disease including lung cancer - non-deterministic, but rather informing further early diagnostic, disease detection, and preventive efforts. These speculations, of course, require considerably more extensive cross-sectional and prospective testing.

In summary, non-invasive access of lower airway tissues for DNA methylation studies appears achievable. Our work demonstrates that DNA methylation in EBC is detectable, can be comprehensively mapped, and in piloting a small number of genes, shows some signal that correlates with tobacco exposure, and perhaps with case-control status. If further characterized and anatomically validated, the approach could help facilitate the non-invasive provision of components of human lung epithelia for epigenetic studies of lung cancer and other lung disease pathogenesis and risk assessment.

## Conclusion

Our results suggest that DNA methylation in exhaled breath condensate is detectable, and in pilot work shows some correlation with smoking and lung cancer case-control status.

## List of abbreviations

EBC: exhaled breath condensate; MSP: Methylation specific PCR; tBGS: tag-modified bisulfite genomic DNA sequencing.

## Competing interests

All four authors have no competing commercial interests. A patent application at USPTO is pending on the tBGS methylation assay.

## Authors' contributions

WH carried out the EBC DNA methylation laboratory studies, and drafted the manuscript. TW and AAR performed the statistical analysis. SDS conceived of EBC methylation, designed the study, aided technical trouble shooting, helped perform the statistical analysis, and drafted and edited the manuscript. All authors read and approved the final manuscript.

## References

[B1] Alberg AJ Ford JG Samet JM Epidemiology of lung cancer: ACCP evidence-based clinical practice guidelines Chest 2007 132 2 29S 55S 10.1378/chest.07-1347 17873159

[B2] Johnson FL Turic B Kemp R Palcic B Sussman R Voelker KG Robinette E Improved diagnostic sensitivity for lung cancer using an automated quantitative cytology system and uridine 5'-triphosphate-induced sputum specimens Chest 2004 125 157S 158S 10.1378/chest.125.5_suppl.157S-a 15136486

[B3] Varella-Garcia M Kittelson J Schulte AP Vu KO Wolf HJ Zeng C Hirsch FR Byers T Kennedy T Miller YE Keith RL Franklin WA Multi-target interphase fluorescence in situ hybridization assay increases sensitivity of sputum cytology as a predictor of lung cancer Cancer Detect Prev 2004 28 244 251 10.1016/j.cdp.2004.04.007 15350627

[B4] Helmig S Schneider J Oncogene and tumor-suppressor gene products as serum biomarkers in occupational-derived lung cancer Expert Rev Mol Diagn 2007 7 555 568 10.1586/14737159.7.5.555 17892364

[B5] Yildiz PB Shyr Y Rahman JS Wardwell NR Zimmerman LJ Shakhtour B Gray WH Chen S Li M Roder H Liebler DC Bigbee WL Siegfried JM Weissfeld JL Gonzalez AL Ninan M Johnson DH Carbone DP Caprioli RM Massion PP Diagnostic accuracy of MALDI mass spectrometric analysis of unfractionated serum in lung cancer J Thorac Oncol 2007 2 893 901 10.1097/JTO.0b013e31814b8be7 17909350 PMC4220686

[B6] Maciel CM Junqueira M Paschoal ME Kawamura MT Duarte RL Carvalho MG Domont GB Differential proteomic serum pattern of low molecular weight proteins expressed by adenocarcinoma lung cancer patients J Exp Ther Oncol 2005 5 31 38 16416599

[B7] Kikuchi T Carbone DP Proteomics analysis in lung cancer: challenges and opportunities Respirology 2007 12 22 28 10.1111/j.1440-1843.2006.00957.x 17207021

[B8] Diederich S CT screening for lung cancer Cancer Imaging 2008 8 Suppl A S24 S26 10.1102/1470-7330.2008.9005 18852077 PMC2582499

[B9] Aisner J CT screening for lung cancer: are we ready for wide-scale application? Clin Cancer Res 2007 13 4951 4953 10.1158/1078-0432.CCR-07-0315 17785542

[B10] Lichy MP Aschoff P Plathow C Stemmer A Horger W Mueller-Horvat C Steidle G Horger M Schafer J Eschmann SM Kiefer B Claussen CD Pfannenberg C Schlemmer HP Tumor detection by diffusion-weighted MRI and ADC-mapping--initial clinical experiences in comparison to PET-CT Invest Radiol 2007 42 605 613 10.1097/RLI.0b013e31804ffd49 17700275

[B11] Blanchon T Brechot JM Grenier PA Ferretti GR Lemarie E Milleron B Chagué D Laurent F Martinet Y Beigelman-Aubry C Blanchon F Revel MP Friard S Rémy-Jardin M Vasile M Santelmo N Lecalier A Lefébure P Moro-Sibilot D Breton JL Carette MF Brambilla C Fournel F Kieffer A Frija G Flahault A Baseline results of the Depiscan study: a French randomized pilot trial of lung cancer screening comparing low dose CT scan (LDCT) and chest X-ray (CXR) Lung Cancer 2007 58 50 58 10.1016/j.lungcan.2007.05.009 17624475

[B12] Bach PB Silvestri GA Hanger M Jett JR Screening for lung cancer: ACCP evidence-based clinical practice guidelines Chest 2007 132 2 69S 77S 10.1378/chest.07-1349 17873161

[B13] Belinsky SA Klinge DM Dekker JD Smith MW Bocklage TJ Gilliland FD Crowell RE Karp DD Stidley CA Picchi MA Gene promoter methylation in plasma and sputum increases with lung cancer risk Clin Cancer Res 2005 11 6505 6511 10.1158/1078-0432.CCR-05-0625 16166426

[B14] Belinsky SA Liechty KC Gentry FD Wolf HJ Rogers J Vu K Haney J Kennedy TC Hirsch FR Miller Y Franklin WA Herman JG Baylin SB Bunn PA Byers T Promoter hypermethylation of multiple genes in sputum precedes lung cancer incidence in a high-risk cohort Cancer Res 2006 66 3338 3344 10.1158/0008-5472.CAN-05-3408 16540689

[B15] Hartung TK Maulu A Nash J Fredlund VG Suspected pulmonary tuberculosis in rural South Africa--sputum induction as a simple diagnostic tool? S Afr Med J 2002 92 455 458 12146131

[B16] Kharitonov SA Barnes PJ Exhaled biomarkers Chest 2006 130 1541 1546 10.1378/chest.130.5.1541 17099035

[B17] Barnes PJ Chowdhury B Kharitonov SA Magnussen H Page CP Postma D Saetta M Pulmonary biomarkers in chronic obstructive pulmonary disease Am J Respir Crit Care Med 2006 174 6 14 10.1164/rccm.200510-1659PP 16556692

[B18] Gessner C Kuhn H Toepfer K Hammerschmidt S Schauer J Wirtz H Detection of p53 gene mutations in exhaled breath condensate of non-small cell lung cancer patients Lung Cancer 2004 43 215 222 10.1016/j.lungcan.2003.08.034 14739043

[B19] Carpagnano GE Foschino-Barbaro MP Mule G Resta O Tommasi S Mangia A Carpagnano F Stea G Susca A Di Gioia G De Lena M Paradiso A 3p microsatellite alterations in exhaled breath condensate from patients with non-small cell lung cancer Am J Respir Crit Care Med 2005 172 738 744 10.1164/rccm.200503-439OC 15947287

[B20] Carpagnano GE Foschino-Barbaro MP Spanevello A Resta O Carpagnano F Mule G Pinto R Tommasi S Paradiso A 3p microsatellite signature in exhaled breath condensate and tumor tissue of patients with lung cancer Am J Respir Crit Care Med 2008 177 337 341 10.1164/rccm.200707-1136OC 17962633

[B21] Han W Cauchi S Herman JG Spivack SD DNA methylation mapping by tag-modified bisulfite genomic sequencing Anal Biochem 2006 355 50 61 10.1016/j.ab.2006.05.010 16797472

[B22] Reik W Stability and flexibility of epigenetic gene regulation in mammalian development Nature 2007 447 425 432 10.1038/nature05918 17522676

[B23] Feinberg AP Phenotypic plasticity and the epigenetics of human disease Nature 2007 447 433 440 10.1038/nature05919 17522677

[B24] Herman JG Baylin SB Gene silencing in cancer in association with promoter hypermethylation N Engl J Med 2003 349 2042 2054 10.1056/NEJMra023075 14627790

[B25] Rakyan VK Hildmann T Novik KL Lewin J Tost J Cox AV Andrews TD Howe KL Otto T Olek A Fischer J Gut IG Berlin K Beck S DNA methylation profiling of the human major histocompatibility complex: a pilot study for the human epigenome project PLoS Biol 2004 2 e405 10.1371/journal.pbio.0020405 15550986 PMC529316

[B26] Lewin J Schmitt AO Adorjan P Hildmann T Piepenbrock C Quantitative DNA methylation analysis based on four-dye trace data from direct sequencing of PCR amplificates Bioinformatics 2004 20 3005 3012 10.1093/bioinformatics/bth346 15247106

[B27] Cox DR Snell EJ Analysis of Binary Data 1989 Second Chapman and Hall/CRC. Boca Raton

[B28] Paulson AS Delehanty TA Heiner KW, Sacher RS, Wilkinson JW Sensitivity Analysis in Experimental Design. In Computer Science and Statistics: Proceedings of the 14th Symposium on the Interface 1982 Springer-Verlag, New York 52 57

[B29] Belinsky SA Palmisano WA Gilliland FD Crooks LA Divine KK Winters SA Grimes MJ Harms HJ Tellez CS Smith TM Moots PP Lechner JF Stidley CA Crowell RE Aberrant promoter methylation in bronchial epithelium and sputum from current and former smokers Cancer Res 2002 62 2370 2377 11956099

[B30] Pulling LC Vuillemenot BR Hutt JA Devereux TR Belinsky SA Aberrant promoter hypermethylation of the death-associated protein kinase gene is early and frequent in murine lung tumors induced by cigarette smoke and tobacco carcinogens Cancer Res 2004 64 3844 3848 10.1158/0008-5472.CAN-03-2119 15172992

[B31] Schafer BW Emerging roles for PAX transcription factors in cancer biology Gen Physiol Biophys 1998 17 211 224 9834843

[B32] Bock C Walter J Paulsen M Lengauer T Inter-individual variation of DNA methylation and its implications for large-scale epigenome mapping Nucleic Acids Res 2008 36 e55 10.1093/nar/gkn122 18413340 PMC2425484

[B33] Bernstein BE Meissner A Lander ES The mammalian epigenome Cell 2007 128 669 681 10.1016/j.cell.2007.01.033 17320505

[B34] Bock C Lengauer T Computational epigenetics Bioinformatics 2008 24 1 10 10.1093/bioinformatics/btm546 18024971

